# Serum cholinesterase: a potential assistant biomarker for hand, foot, and mouth disease caused by enterovirus 71 infection

**DOI:** 10.1186/s40249-016-0124-y

**Published:** 2016-03-29

**Authors:** Bang-Ning Cheng, Yu-Lian Jin, Bi-Quan Chen, Li-Yan Zhu, Zi-Cheng Xu, Tao Shen

**Affiliations:** Clinical Laboratory Center, Anhui Provincial Children’s Hospital, Hefei, 230051 China; Department of Infectious Diseases, Anhui Provincial Children’s Hospital, Hefei, 230051 China; Department of Microbiology and Infectious Disease Center, School of Basic Medical Sciences, Peking University, Beijing, 100191 China

**Keywords:** Serum cholinesterase, Hand, foot, and mouth disease, Enterovirus 71, Coxsackievirus A16, Children, Biochemical marker

## Abstract

**Background:**

Hand, foot, and mouth disease (HFMD) caused by enterovirus 71 (EV71) is a potentially life-threatening infectious disease that commonly occurs in children. Diagnosis of HFMD caused by EV71 largely depends on clinical manifestations and rare serological biomarkers used to identify children suffering from HFMD. Serum cholinesterase (SChE) activity has frequently been reported as a potential biomarker for solid central nervous system tumors, chronic heart failure, and liver cirrhosis. However, its potential value in the diagnosis of neurotropic virus infections, such as HFMD caused by EV71, remains to be determined.

**Findings:**

In our study, 220 children hospitalized with HFMD caused by EV71, 34 inpatients infected with coxsackievirus A16 (CVA16), and 43 undefined enterovirus-infected HFMD inpatients were recruited at the Anhui Provincial Children’s Hospital between January 2011 and December 2012. SChE activity was measured. The non-parametric Mann–Whitney *U* test showed that SChE activity in children diagnosed with HFMD caused by EV71 was significantly higher than in healthy controls (*p* < 0.001), as well as in children with upper respiratory tract infections (*p* = 0.011), bronchopneumonia (*p* < 0.001), septicemia (*p* < 0.001), amygdalitis (*p* < 0.001), and appendicitis (*p* < 0.001). In addition, higher SChE activity was observed in male inpatients with HFMD caused by EV71 (47.7 % positivity) compared to female inpatients (26.1 % positivity) (chi-square test, *p* = 0.002). In our study, no significant differences in SChE levels were observed among different ages (up to 120 months) (*r* = -0.112, *p* > 0.05). An important finding was that SChE activity declined in the recovery phase of HFMD caused by EV71 compared to the acute phase (*p* < 0.001).

**Conclusions:**

Elevated SChE activity was observed in patients with severe HFMD caused by EV71. Therefore, SChE might be a potential assistant biomarker for the diagnosis of HFMD caused by EV71 in children.

**Electronic supplementary material:**

The online version of this article (doi:10.1186/s40249-016-0124-y) contains supplementary material, which is available to authorized users.

## Multilingual abstracts

Please see Additional file 1 for translation of the abstract into the six official working languages of the United Nations.

## Findings

Hand, foot, and mouth disease (HFMD) is a potentially life-threatening infectious disease commonly found in children. In China, 1,828,377 HFMD cases were diagnosed in 2013; among these, 252 cases resulted in death, according to the report from the national Health and Family Commission of China [1]. The main pathogens of HFMD are enteroviruses. In recent years, most cases of HFMD in mainland China were caused by enterovirus 71 (EV71) [2–4], followed by coxsackievirus A16 (CVA16), as well as other enterovirus serotypes. The diagnosis of HFMD caused by EV71 largely depends on the former’s clinical manifestations, such as fever; vesicular rashes on palms, soles, and mouth; and, in severe cases, central nervous system (CNS) involvement [3, 5, 6]. Certain serological biomarkers are helpful for early diagnosis, timely surveillance, and appropriate medical treatment of HFMD, in order to avoid disease progression and death. Although some reports indicate that serum levels of cytokines, such as IL-2, IL-4, IL-10, IFN-γ, GM-CSF, and TNF-α, may be independent risk factors for early medical intervention [7, 8], at present there are no reliable and easily-operated biomarkers that could be used to assist diagnosis of HFMD in clinical practice.

Serum cholinesterase (SChE) is an enzyme synthesized by the liver. It has been reported that the enzyme’s activity changes under certain disease conditions such as liver malfunction, obesity, diabetes, inflammation, and leptospiral infection [9, 10]. Serum cholinesterase is a routine parameter included in biochemical panels in clinical laboratory settings. Therefore, it could serve as a potential biomarker for clinical diagnosis. To date, characteristics of SChE activity have not been reported in HFMD, and its potential diagnostic value for HFMD is also unclear. Therefore in this study, we measured SChE activity in HFMD patients and evaluated its potential diagnostic value.

A total of 220 children hospitalized with HFMD caused by EV71, 34 inpatients infected with CVA16, and 43 inpatients infected with pan-enteroviruses (members of the enterovirus genus, PE) were recruited at the Anhui Provincial Children’s Hospital between January 2011 and December 2012. All patients were aged one month to 10 years and exhibited complications, with varying degrees of CNS involvement, that caused symptoms such as myoclonus, ataxia, nystagmus, oculomotor palsies, and bulbar palsy in various combinations; and/or respiratory distress with tachycardia, tachypnea, and rales, with or without frothy sputum. All HFMD cases were diagnosed according to China’s Ministry of Health guidelines [11]. Patient demographics, clinical symptoms, and major complications were collected retrospectively from the patients’ medical histories. Whole blood samples from all participants were collected and used for routine blood testing and in serum biochemical parameter tests. Stool specimens were collected from children diagnosed with HFMD and kept at -80 °C until examined for the presence of enteroviruses by reverse transcription polymerase chain reaction (RT-PCR). Viral RNA was extracted from the supernatant of 10 % (V/V) stool specimens using the QIAamp Viral RNA Mini Kit (Qiagen, Hilden, Germany) and subjected to RT-PCR according to the manufacturer’s instructions (DAAN Gene, Guangzhou, China) [12–14]. The RT-PCR kit specifically detected PE, EV71, and CVA16. The classification of EV71, CVA16, and PE depended on the analysis of real-time data. In addition, a total of 155 age-matched HFMD-negative inpatient children diagnosed with upper respiratory tract infections (URTIs) (*n* = 11), bronchopneumonia (BP) (*n* = 43), septicemia (*n* = 27), amygdalitis (*n* = 7), and appendicitis (*n* = 18), as well as 80 age-matched healthy children, were used as controls. The study was approved by the institutional review board at Peking University Health Science Center and the Anhui Provincial Children’s Hospital. Because the specimens were collected during the normal course of patient care, no informed consent was required, according to the Ethics Committee.

Serum samples were collected from clotted blood and detection of serum biochemical parameters was carried out within two hours of sample separation. Serum cholinesterase activity was determined using an assay, with butyrylthiocholine as the substrate. The analyses were done using Leadman reagent kits (Leadman, Beijing, China) on a Hitachi 7600 Automatic Biochemical Analyzer (Hitachi Ltd., Tokyo, Japan). The normal range of serum SChE was between 4500 and 13,000 IU/L, using the enzyme velocity method.

**Fig. 1:**
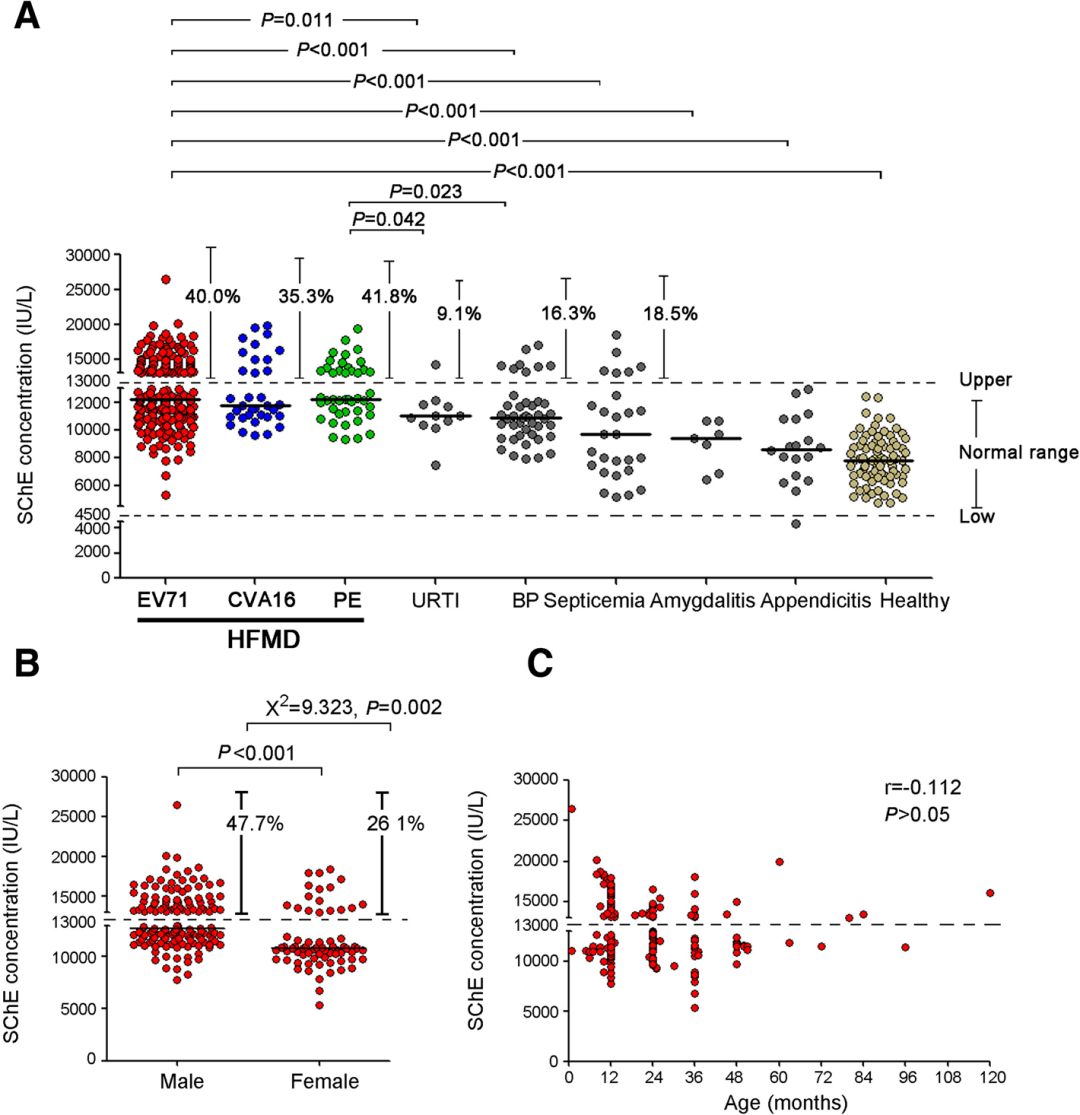
Characteristics of SChE activity in inpatient children infected with HFMD caused by EV71. **a** SChE activity in 220 inpatient children infected with HFMD caused by EV71, 34 patients infected with CVA16, and 43 patients infected with PE was compared with SChE activity in healthy children (*n* = 80) and HFMD-negative children diagnosed with other common diseases including URTIs (*n* = 11), BP (*n* = 43), septicemia (*n* = 27), amygdalitis (*n* = 7), and appendicitis (*n* = 18). **b** Higher SChE activity was observed in inpatient males infected with HFMD caused by EV71 compared to female inpatients. **c** No significant differences were observed in elevated SChE levels among EV71-infected patients of different ages

**Fig. 2:**
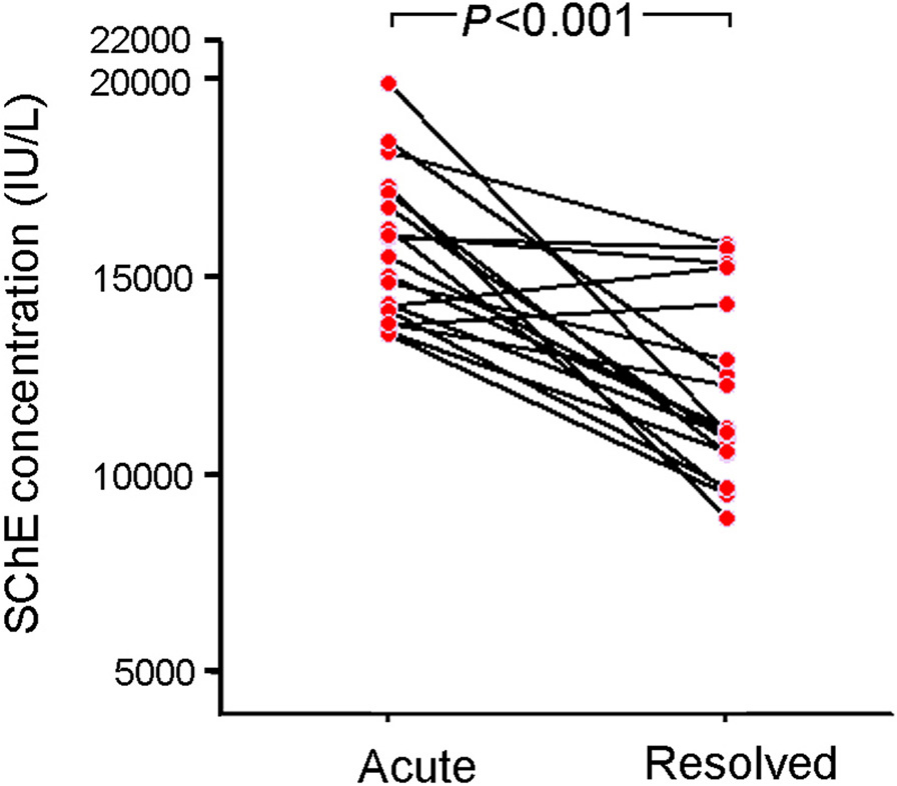
The higher SChE concentration in 19 patients with HFMD caused by EV71 that was observed in the acute phase of the disease declined in the recovery phase

In our study, increased SChE activity, defined as higher than 13,000 IU/L, was observed in 40 % of children with HFMD caused by EV71 (see Fig. 1a). The non-parametric Mann–Whitney *U* test showed that SChE activity was significantly higher in children suffering from HFMD caused by EV71 than in healthy controls (0 % positivity, *p* < 0.001), as well as children with URTIs (9.1 % positivity, *p* = 0.011), BP (16.3 % positivity, *p* < 0.001), septicemia (18.5 % positivity, *p* < 0.001), amygdalitis (0 % positivity, *p* < 0.001), and appendicitis (0 % positivity, *p* < 0.001). No significant differences in SChE activity were observed among HFMD patients infected with EV71, CVA16 (35.3 % positivity), and PE (41.8 % positivity). In addition, higher SChE activity was observed in male HFMD inpatients (47.7 % positivity) compared to female inpatients (26.1 % positivity) (Student’s *t*-test, *p* < 0.001; chi-square test, *p* = 0.002) (see Fig. 1b). Most cases of HFMD caused by EV71 occurred in children below the age of two, and particularly in infants under one year of age (see Fig. 1c). However, we did not observe a significant difference in elevated SChE levels among different ages (up to 120 months) (*r* = -0.112, *P* > 0.05; see Fig. 1c). Furthermore, as shown in Fig. 2, after tracking SChE activity in 19 patients suffering from acute HFMD for more than two months, we found that serum SChE activity declined during the recovery phase compared to the acute phase of the disease (*p* < 0.001).

Cholinesterase consists of two members, i.e., acetylcholinesterase (AChE) and SChE [15]. Acetylcholinesterase is mainly found in erythrocytes, and in the central and peripheral nervous systems. Its main role is to catalyze the hydrolysis of the neurotransmitter acetylcholine into choline and acetic acid, a termination process necessary for neuronal transmission after activation. In contrast to AChE, SChE—also known as plasma cholinesterase, but yrylcholinesterase, or acetylcholine acylhydrolase—is synthesized primarily in the liver and distributed to circulating fluids. Serum cholinesterase serves mostly as a nonspecific esterase to cleave various esters, even those with bulkier acyl moieties [16]. Recently, SChE has been reported as a potential biomarker [17] for solid CNS tumors [18], chronic heart failure [19], liver cirrhosis [20, 21], parasympathetic dysfunction, and inflammation-related diseases [22], although the underlying mechanisms have yet not been clearly identified. To our knowledge, its potential value to diagnose neurotropic virus infections, such as EV71-associated HFMD, is currently unclear.

Our study had some limitations. Because the majority of the inpatient children suffering from HFMD that we studied were infected with EV71, our study failed to recruit a manageable number of patients infected with other enterovirus serotypes or other infectious diseases. Our study may indicate that higher SChE activity in HFMD inpatient children is not an EV71-specific phenomenon, but is also present in HFMD patients infected with other enteroviruses. This phenomenon should be verified in a study with a much larger cohort of non-EV71-infected HFMD patients. Also, it should be further analyzed if CNS symptoms complicated with HFMD caused by EV71 are associated with higher SChE activity.

Despite these limitations, our study is the first to report that SChE activity in peripheral blood is higher in HFMD children than in children diagnosed with other common diseases and healthy children. This clearly demonstrates that SChE activity can be used as a potential assistant biomarker for identifying children suffering from HFMD caused by EV71.

## Additional file

Additional file 1: Multilingual abstracts in the six official working languages of the United Nations. (PDF 427 kb)

## Abbreviations

AChE: acetylcholinesterase
BP: bronchopneumonia
CNS: central nervous system
CVA16: coxsackievirus A16
EV71: enterovirus 71
HFMD: hand, foot, and mouth disease
PE: enterovirus genus
RT-PCR: reverse transcriptase polymerase chain reaction
SChE: serum cholinesterase
URTI: upper respiratory tract infection
