# Supplementary material for: Serum cholinesterase: a potential assistant biomarker for hand, foot, and mouth disease caused by enterovirus 71 infection
Source: Infect Dis Poverty. 2016 Mar 29;5:27. doi: 10.1186/s40249-016-0124-y (PMC4812613; doi:10.1186/s40249-016-0124-y)
Supplement: Additional file 1: — Multilingual abstracts in the six official working languages of the United Nations. (PDF 427 kb) [file 40249_2016_124_MOESM1_ESM.pdf]

Translation of the abstract into the six official working languages of the United Nations

إنزيم الكولين المصل: مساعد محتمل للعلامات البيولوجية لأمراض اليد والقدم و الفم الناجمة عن عدوى الفيروس المعوي 71

بانغننغ تشنغ، يوليان جن، بيكوان تشن، لي يان تسو، تسي تشنغ شو، وتاو شين

الملخص

الخلفية: مرض اليد والقدم والفم (الحمى الثلاثية) الذي يسببه الفيروس المعوي 71 (EV71) هو مرض معدي يهدد الحياة وهو يحدث عادة في الأطفال. يعتمد تشخيص مرض الحمى الثلاثية الناتجة عن EV71 إلى حد كبير على المظاهر السريرية والمؤشرات الحيوية المصلية النادرة المستخدمة لتحديد الأطفال الذين يعانون من مرض الحمى الثلاثية. وكثيراً ما ذكر نشاط إنزيم الكولين المصل (SChE) كعلامة بيولوجية محتملة للأورام الصلبة للجهاز العصبي المركزي، وفشل القلب المزمن، وتليف الكبد. ومع ذلك، فقيمته المحتملة في تشخيص عدوى الفيروس الموجه للعصب، مثل الحمى الثلاثية الناتجة عن EV71، لا يزال يتعين تحديدها.

النتائج: في دراستنا ، دخل 220 طفلاً المستشفى بمرض الحمى الثلاثية الناتجة عن EV71، منهم 34 مريضاً مصاباً بفيروس coxsackievirusA16 (CVA16)، و 43 مريضاً بالحمى الثلاثية مصابون بفيروس معوي غير محدد في مستشفى مقاطعة انهوى للأطفال بين يناير 2011 وديسمبر 2012. وقد تم قياس نشاط المصل SChE. أظهر الاختبار اللا حدودي Mann-Whitney U أن نشاط المصل SChE في الأطفال الذين تم تشخيصهم بمرض الحمى الثلاثية الناتجة عن EV71 كان أعلى بكثير مما كان عليه في الاصحاء (P <0.001)، وكذلك في الأطفال الذين يعانون من التهابات الجهاز التنفسي العلوي (p=0.011) ، القصبي الرئوي (P <0.001)، تسمم الدم (P <0.001)، التهاب اللوزتين (P <0.001)، والتهاب الزائدة الدودية (P <0.001). بالإضافة إلى ذلك، لوحظ ارتفاع نشاط SChE في المرضى المنومين من الذكور المصابين بمرض الحمى الثلاثية الناتجة عن EV71(47.7%) إيجابية) مقارنة مع المرضى المنومين الإناث (26.1٪ إيجابية) (اختبار مربع تشي ، p=0.002). في دراستنا، لم يلاحظ أي اختلافات كبيرة في مستويات SChE بين مختلف الأعمار (تصل إلى 120 شهراً) (r=0.112، P> 0.05). كان الاكتشاف المهم هو أن نشاط SChE انخفض في مرحلة التعافي من الحمى الثلاثية الناتجة عن EV71 بالمقارنة مع المرحلة الحادة (P <0.001).

الاستنتاجات: لوحظ ارتفاع نشاط SChE في المرضى الذين يعانون من مرض الحمى الثلاثية الحادة الناتجة عن EV71. ولذلك، قد يكون SChE مساعداً محتملاً للعلامات البيولوجية لتشخيص مرض الحمى الثلاثية الناتجة عن EV71 في الأطفال.

Translated from English version into Arabic by Free bird, through

血清胆碱酯酶：肠道病毒 71 型感染所致手足口病的潜在辅助生物标记物

Bangning Cheng, Yulian Jin, Biquan Chen, Liyan Zhu, Zicheng Xu and Tao Shen

摘要

引言：肠道病毒 71 型（EV71）所致手足口病（HFMD）是一种常见的儿童传染病，对患儿的生命造成潜在的威胁。主要根据临床症状来诊断 EV71 所致 HFMD，可用于诊断 HFMD 患儿的血清学生物标记物极少。据报道，血清胆碱酯酶（SChE）活性可作为中枢神经系统肿瘤、慢性心力衰竭、肝硬化的潜在生物标志物。然而，SChE 在神经病毒感染（如 EV71 所致手足口病）诊断中的潜在价值仍有待确定。

发现：2011 年 1 月至 2012 年 12 月，安徽省儿童医院共收治 297 例 HFMD 住院患儿，其中 220 例为 EV71 感染，34 例感染柯萨奇病毒 A16 型（CVA16）感染患者和 43 例为未确诊肠道病毒感染患者。测定 SChE 活性。非参数检验 Mann–WhitneyU 检验结果表明，EV71 所致 HFMD 患儿的 SChE 活性显著高于健康对照

组（*P*<0.001），以及以下患儿，如上呼吸道感染（*P*=0.011）、支气管肺炎（*P*<0.001）、败血病（*P*<0.001）、扁桃体炎（*P*<0.001）和阑尾炎（*P*<0.001）。此外， EV71 所致 HFMD 住院患儿的 SChE 活性，且男童的显著高于的（47.7 %阳性）高于女童（26.1%阳性）（卡方检验，*P*=0.002）。在本研究中，不同年龄组（最大的患儿 10 岁）人群的 SChE 水平差异无统计学意义（r=-0.112，*P*>0.05）。本研究的一个重要发现是相较于急性期，在 EV71 所致 HFMD 的恢复期 SChE 活性较低（*P*<0.001）。

结论：EV71 所致 HFMD 重症患者的 SChE 活性较高。因此，SChE 或可成为诊断 EV71 所致 HFMD 患儿的一个潜在辅助生物标志物。

Translated from English version into Chinese by Chen Jin, edited by Yang Pin, through

CHINESE CENTER FOR DISEASE CONTROL AND PREVENTION
NATIONAL INSTITUTE OF PARASITIC DISEASES

Cholinestérase sérique : un biomarqueur potentiel de la maladie pieds-mains-bouche causée par un infection à l'entérovirus 71

Bangning Cheng, Yulian Jin, Biquan Chen, Liyan Zhu, Zicheng Xu et Tao Shen

Résumé

Contexte : La maladie pieds-mains-bouche causée par l'entérovirus 71 (EV71) est une maladie infectieuse potentiellement mortelle touchant généralement les enfants. Le diagnostic de la maladie pieds-mains-bouche causée par l'EV71 dépend largement de manifestations cliniques ainsi que de la présence de biomarqueurs sérologiques rares utilisés pour identifier les enfants atteints de cette maladie. L'activité de la cholinestérase sérique (SChE) a souvent été désignée comme un biomarqueur potentiel de tumeurs solides du système nerveux central, d'insuffisance cardiaque chronique et de cirrhose hépatique. Sa valeur potentielle dans le diagnostic d'infections virales neurotropiques, telles que la maladie pieds-mains-bouche causée par l'EV71, reste néanmoins à déterminer.

Observations : Notre étude a regroupé 220 enfants hospitalisés et atteints d'une maladie pieds-mains-bouche causée par l'EV71, 34 patients hospitalisés infectés par le virus Coxsackie A16 (CVA16) et 43 patients hospitalisés atteints d'une maladie pieds-mains-bouche causée par un entérovirus non déterminé de l'Hôpital pour enfants de la Province de Anhui entre janvier 2011 et décembre 2012. L'activité de la SChE sérique a été mesurée. Le test de Wilcoxon-Mann-Whitney non paramétrique a démontré que l'activité de la SChE sérique parmi des enfants chez lesquels une maladie pieds-mains-bouche causée par l'EV71 avait été diagnostiquée était nettement plus élevée que chez les contrôles sains (*p*<0,001) ainsi que chez des enfants atteints d'infections des voies respiratoires supérieures (*p*=0,011), de bronchopneumonie (*p*<0,001), de septicémie (*p*<0,001), d'amygdalite (*p*<0,001) et d'appendicite (*p*<0,001). De plus, une activité plus élevée de la SChE a été observée chez des patients hospitalisés atteints d'une maladie pieds-mains-bouche causée par l'EV71 (47,7 % de positivité) par rapport à des patientes hospitalisées (26,1 % de positivité) (test du chi carré, *p*=0.002). Dans le cadre de notre étude, aucune différence significative en termes de taux de SChE n'a été observée parmi les différentes classes d'âge (jusqu'à 120 mois) (r=-0,112, *p*>0,05). Il convient de remarquer que l'activité de la SChE diminuait au cours de la phase de guérison de la maladie pieds-mains-bouche causée par l'EV71 par rapport à la phase aiguë (*p*<0,001).

Conclusions : Une hausse de l'activité de la SChE a été observée chez des patients atteints d'une maladie pieds-mains-bouche sévère causée par l'EV71. La SChE pourrait donc constituer un biomarqueur potentiel pour le

diagnostic de la maladie pieds-mains-bouche causée par l'EV71 chez les enfants.

Translated from English version into French by eric ragu, through

Холинэстераза сыворотки: потенциальный вспомогательный биомаркер заболевания кистей рук, стоп и полости рта, вызываемого энтеровирусом 71

Баннин Чен, Юлянь Джин, Бичуан Чен, Лиян Жу, Цзычен Су, и Тао Шен

Краткий обзор статьи

Исходные данные: Заболевание кистей рук, стоп и полости рта (ЗКСП), вызываемое энтеровирусом 71 (EV71) является потенциально опасным инфекционным заболеванием, которое часто встречается у детей. Диагностика ЗКСП, вызываемого EV71, во многом зависит от клинических проявлений и редких серологических биомаркеров, используемых для выявления детей, страдающих от ЗКСП. Активность холинэстеразы сыворотки (SChE) часто отражается в качестве потенциального биомаркера плотной опухоли центральной нервной системы, хронической сердечной недостаточности и цирроза печени. Тем не менее, ее потенциальное значение для диагностики нейротропных вирусных инфекций, таких как ЗКСП, вызываемого EV71, еще предстоит определить.

Полученные результаты: Наше исследование охватывает 220 детей, госпитализированных с ЗКСП, вызванным EV71, 34 пациентов, инфицированных вирусом Коксаки A16 (CVA16), и 43 пациента с неопределенной энетеровирусной инфекцией ЗКСП – пациенты поступили в детскую больницу провинции Аньхой в период между январем 2011 г. и декабрем 2012 г. В ходе исследования измерялась активность сыворотки SChE. Непараметрический U-тест Манна-Уитни показал, что активность сыворотки SChE у детей с диагнозом ЗКСП, вызванным EV71, значительно выше, чем у здоровых представителей контрольной группы (p<0.001), а также у детей с инфекциями верхних дыхательных путей (p=0.011), бронхопневмонией (p<0.001), сепсисом (p<0.001), тонзиллитом (p<0.001), и аппендицитом (p<0.001). Кроме того, повышенная активность SChE наблюдалась у госпитализированных пациентов мужского пола с ЗКСП, вызванным EV71 (47.7% положительный результат) по сравнению с пациентами женского пола (26.1% положительный результат) (критерий хи-квадрат, p=0.002). В нашем исследовании не наблюдалось существенных различий уровня SChE в разных возрастных группах (до 120 месяцев) (r=-0.112, p>0.05). Важным выводом стало то, что активность SChE снижалась на стадии выздоровления от ЗКСП, вызванного EV71, по сравнению с острой фазой (p<0.001).

Заключение: Повышенная активность SChE наблюдалась у пациентов с тяжелой формой ЗКСП, вызванного EV71. Поэтому SChE может быть потенциальным вспомогательным биомаркером для диагностики ЗКСП, вызванного у детей вирусом EV71.

Translated from English version into Russian by Jekaterina Merkuljeva, through

Colinesterasa sérica: un biomarcador potencial para la enfermedad de manos, pies y boca provocados por la infección de enterovirus 71

Bangning Cheng, Yulian Jin, Biquan Chen, Liyan Zhu, Zicheng Xu y Tao Shen

Resumen

Información de referencia: La enfermedad de manos, pies y boca (hand, foot, and mouth disease, HFMD) provocada por el enterovirus 71 (EV71) es una enfermedad infecciosa potencialmente mortal que afecta generalmente a niños. El diagnóstico de HFMD provocado por el EV71 depende en gran parte de manifestaciones clínicas y de biomarcadores serológicos raros utilizados para identificar a los niños que sufren HFMD. Se ha señalado a menudo la actividad de colinesterasa sérica (SChE) como biomarcador potencial de tumores sólidos en el sistema nervioso central, insuficiencia cardíaca crónica y cirrosis hepática. No obstante, sigue pendiente de determinar su valor potencial en el diagnóstico de infecciones de virus neurotrópicos, como HFMD provocada por EV71.

Hallazgos: En nuestro estudio participaron 220 niños hospitalizados con HFMD provocada por EV71, 34 pacientes infectados con coxsackievirus A16 (CVA16) y 43 pacientes de HFMD infectados con un enterovirus indefinido, todos ellos el Hospital Infantil Provincial de Anhui, entre enero de 2011 y diciembre de 2012. Se midió la actividad de SChE sérica. La prueba Mann–Whitney U no paramétrica indicó que la actividad de SChE sérica en niños diagnosticados con HFMD provocada por EV71 era significativamente más alta que en controles sanos (p<0,001), así como en niños con infecciones en el tracto respiratorio superior (p=0,011), bronconeumonía (p<0,001), septicemia (p<0,001), amigdalitis (p<0,001) y apendicitis (p<0,001). Además se observó una mayor actividad de SChE sérica en pacientes varones con HFMD provocada por EV71 (positividad del 47,7%) comparada con pacientes hembras (positividad del 26,1%) (test de chi cuadrado, p=0,002). En nuestro estudio no se observaron diferencias significativas en los niveles de SChE entre diferentes edades (hasta 120 meses) (r=-0,112, p>0,05). Un importante hallazgo fue que la actividad de SChE descendía en la fase de recuperación de HFMD provocada por EV71 si se compara con la fase aguda (p<0,001).

Conclusiones: Se observe una elevada actividad de SChE en pacientes con HFMD severa provocada por EV71. Por tanto, SChE podría ser un biomarcador potencial para el diagnóstico de HFMD provocada por EV71 en niños.

Translated from English version into Spanish by Sergio Lorenzi, through

TRANSLATORS WITHOUT BORDERS
